# Urbanization reduces gene flow but not genetic diversity of stream salamander populations in the New York City metropolitan area

**DOI:** 10.1111/eva.13025

**Published:** 2020-06-12

**Authors:** Nicole A. Fusco, Ellen Pehek, Jason Munshi‐South

**Affiliations:** ^1^ Department of Biology Fordham University Bronx NY USA; ^2^ Natural Resources Group New York City Department of Parks & Recreation New York NY USA

**Keywords:** genetic connectivity, stream salamanders, urbanization

## Abstract

Natural landscape heterogeneity and barriers resulting from urbanization can reduce genetic connectivity between populations. The evolutionary, demographic, and ecological effects of reduced connectivity may lead to population isolation and ultimately extinction. Alteration to the terrestrial and aquatic environment caused by urban influence can affect gene flow, specifically for stream salamanders who depend on both landscapes for survival and reproduction. To examine how urbanization affects a relatively common stream salamander species, we compared genetic connectivity of *Eurycea bislineata* (northern two‐lined salamander) populations within and between streams in an urban, suburban, and rural habitat around the New York City (NYC) metropolitan area. We report reduced genetic connectivity between streams within the urban landscape found to correspond with potential barriers to gene flow, that is, areas with more dense urbanization (roadways, industrial buildings, and residential housing). The suburban populations also exhibited areas of reduced connectivity correlated with areas of greater human land use and greater connectivity within a preserve protected from development. Connectivity was relatively high among neighboring rural streams, but a major roadway corresponded with genetic breaks even though the habitat contained more connected green space overall. Despite greater human disturbance across the landscape, urban and suburban salamander populations maintained comparable levels of genetic diversity to their rural counterparts. Yet small effective population size in the urban habitats yielded a high probability of loss of heterozygosity due to genetic drift in the future. In conclusion, urbanization impacted connectivity among stream salamander populations where its continual influence may eventually hinder population persistence for this native species in urban habitats.

## 1 INTRODUCTION

Urbanization imposes unique pressures on wildlife populations that alter patterns of genetic variation in species occupying urban habitats (Evans, 2010; Johnson & Munshi‐South, 2017; Keyghobadi, 2007; Miles, Rivkin, Johnson, Munshi‐South, & Verrelli, 2019). Reduced genetic connectivity between urban populations is now well‐documented (Delaney, Riley, & Fisher, 2010; Kobayashi, Abe, Tomita, & Matsuki, 2018; Savage et al., 2015; Toczydlowski & Waller, 2019) where changes to gene flow and drift are the most prominent results cited in recent urban evolution literature (Johnson & Munshi‐South, 2017). A review by Miles et al. (2019) concluded that variation in species biology and differences among urban environments leads to a variety of outcomes such as facilitation, reduction, or no effect on gene flow, with a general tendency for urbanization to hinder gene flow. With considerable variation in life history traits, amphibians have the potential for varied responses to anthropogenically altered habitats. Many studies have shown that amphibians have narrow habitat tolerances and are highly vulnerable to pathogens and pollution, making them particularly susceptible to urban disturbance (Becker, Roberto Fonseca, Haddad, Batista, & Prado, 2007; Beebee, 2005; Cushman, 2006). Consequently, these vulnerabilities often cause reduced gene flow between amphibian populations (Cameron, Page, Watling, Hickerson, & Anthony, 2019; Cayuela et al., 2020; Emel & Storfer, 2014; Furman, Scheffers, Taylor, Davis, & Paszkowski, 2016). Stream salamanders have a biphasic lifecycle (i.e., aquatic larval and semiterrestrial adult stages) where they can disperse either along stream branches or overland between branches (Grant, Nichols, Lowe, & Fagan, 2010). Thus, they are particularly prone to both aquatic and terrestrial modifications caused by urbanization (Johansson, Primmer, Sahlsten, & Merilä, 2005; Munshi‐South, Zak, & Pehek, 2013; Pillsbury, Miller, & Miller, 2008).

Landscape features encountered during dispersal fundamentally affect connectivity between populations. Reduced leaf litter depth, soil moisture (Crawford & Semlitsch, 2008), and canopy cover (Cecala, Lowe, & Maerz, 2014) in forest habitats restrict terrestrial microhabitat use by stream salamanders. Alterations to hydrologic characteristics of the stream itself (pH, temperature, Barrett & Price, 2014; conductivity; Willson & Dorcas, 2003) and loss of microhabitats within the stream (substrate composition and embeddedness; Lowe & Bolger, 2002) are known to reduce stream salamander presence, survival, and abundance in urban streams. Greater impervious surface in urban habitats also increases high water flow frequency and magnitude within streams in urban areas (Walsh et al., 2005), thus decreasing stream salamander density (Barrett, Helms, Guyer, & Schoonover, 2010). If potential habitat for dispersal and reproduction for stream salamanders is limited in urban areas, resultant gene flow and genetic variation within populations may also decrease.

Effective dispersal (the movement of individuals between successive breeding sites; Matthysen, 2012; Ronce, 2007) potentially facilitates gene flow (the movement of alleles; Cayuela et al., 2018) within spatially structured populations (Thomas & Kunin, 1999). At the population level, gene flow reduces extinction risk by counteracting the detrimental effects of genetic drift (Frankham, Ballou, & Briscoe, 2002; Kraaijeveld‐Smit, Beebee, Griffiths, Moore, & Schley, 2005). An organism's morphology (e.g., body size), physiological tolerance, and life history traits that alter dispersal rate and distance through the land‐ and streamscape (Ronce & Clobert, 2012) can also affect gene flow patterns. Occupancy of headwater streams by stream salamanders is also influenced by the spatial configuration of the stream itself (Grant, Green, & Lowe, 2009). Urban fragmentation can create barriers within already complicated stream networks, limiting gene flow and creating more severe consequences than in simple linear or two‐dimensional systems (Fagan, 2002).

The spatial distribution of populations within human‐impacted landscapes influences patterns of gene flow and spatial genetic variation. When dispersal distance spatially limits species, populations geographically farther apart will have greater genetic differentiation between them, a pattern known as isolation‐by‐distance (IBD; Wright, 1943). Alternatively, urban habitat degradation in the terrestrial landscape caused by roadways (Fenderson et al., 2014; Serieys, Lea, Pollinger, Riley, & Wayne, 2015), buildings (Beninde, Veith, & Hochkirch, 2015), loss of green space (Spear, Peterson, Matocq, & Storfer, 2005), or dams (Bohling, Starcevich, Von Bargen, & Bailey, 2019) may produce nonpermeable barriers restricting gene flow between populations (isolation‐by‐barrier; IBB; Smouse, Long, & Sokal, 1986). Both IBD and IBB ultimately shape patterns of genetic variation within and between populations. Understanding these drivers can reveal which evolutionary mechanisms have shaped and structured populations over recent generations.

Plethodontids (i.e., lungless salamanders) are the most ubiquitous family of salamanders in northeastern North America and comprise a sizeable proportion of vertebrate biomass in temperate forests (Burton & Likens, 1975). In this study, we explored genetic connectivity for one of the most widespread and common stream‐associated species, the northern two‐lined salamander (*Eurycea bislineata*). This species shows extensive dispersal throughout streams (Bruce, 1986) and can disperse over land (Grant et al., 2010; Lowe, 2014; Miller, Snodgrass, & Gasparich, 2015). For this reason, members of this species are an excellent system for understanding how urbanization affects gene flow in both terrestrial and aquatic habitats. *E. bislineata* is one of the few stream salamanders to occupy highly urbanized areas (Barrett et al., 2010), but the degree to which they maintain genetic connectivity in urban stream networks is currently unknown. Given extensive knowledge on how ecological disturbance negatively affects the presence and abundance of *E. bislineata* in urban habitats (Barrett et al., 2010; Barrett & Price, 2014; Hamer & McDonnell, 2008), we predicted that increasing levels of urbanization will limit gene flow between populations and accelerate the loss of genetic diversity due to drift in urban populations.

Population genetic studies comparing urban and nonurban habitats are necessary to determine the influence of urbanization, where nonurban sites act as a baseline to elucidate whether urbanization is a factor altering gene flow and genetic drift (Miles et al., 2019). Many studies have now shown that urbanization influences the structure of wildlife populations in NYC (Combs, Puckett, Richardson, Mims, & Munshi‐South, 2018; Henger et al., 2019; Munshi‐South et al., 2013; Munshi‐South, Zolnik, & Harris, 2016; Savage et al., 2015), but many of these studies did not compare urban and nonurban populations. Additionally, very few studies have used genomic data to study gene flow in salamanders in general (Murphy, Jones, Price, & Weisrock, 2018). We used reduced representation sequencing (ddRADseq; Peterson, Webber, Kay, Fisher, & Hoekstra, 2012) to generate single nucleotide polymorphisms (SNPs) over thousands of loci across many individuals (Rovelli, Ruiz‐González, & Davoli, 2018) to assess genetic connectivity and levels of genetic variation among *E. bislineata* in an urban, suburban, and rural habitat.

In this study, we aimed to answer the following questions: (1) Do levels of genetic connectivity differ for *E. bislineata* within an urban, a suburban, and a rural habitat? (2) How does connectivity affect genetic diversity across habitats? (3) Do geographic distance (IBD) or barriers (IBB) better explain connectivity within each habitat type? We predicted that individuals within the urban habitat would show greater genetic differentiation between neighboring streams compared to the less‐developed suburban and rural habitats. We also predicted that genetic diversity and effective population size would be lowest in the urban habitat due to a loss of connectivity and highest in the rural habitat. Lastly, we predicted that IBD will structure populations in the suburban and rural stream networks, whereas IBB would be more influential in the urban habitat.

## 2 METHODS

### 2.1 Study species

The northern two‐lined salamander (*E. bislineata*) is a generally abundant stream‐dwelling species throughout its large range, from northern Ontario and Quebec to southern Virginia (Burton & Likens, 1975; Hammerson, 2004; Sever, 1999). Although IUCN Red List categorizes this species as least concern (Hammerson, 2004), some studies show a reduction in local abundance (Petranka, 1998) and density (Pehek & Stanley, 2015) for *E. bislineata* in urban areas. This species shows high occupancy in streams with ample cover objects (Smith & Grossman, 2003) such as cobble (Barr & Babbitt, 2002) and other debris (Ashton & Ashton, 1978), and will use near‐stream terrestrial habitats with high soil moisture, low soil temperatures, and deep leaf litter (Crawford & Semlitsch, 2007). Individuals of this species more often occupy low‐order (headwater) streams and show higher occupancy in branched versus unbranched watershed systems (Grant et al., 2009). Pehek (2007) found *E. bislineata* to be one of the most abundant salamander species in NYC, with recent sightings from 1980 to 2007 across four NYC boroughs (excluding Manhattan where it was recorded only prior to 1979). The highest salamander diversity in NYC recorded at that time was located on Staten Island (Richmond Co.), which also contains the most well‐documented locations for *E. bislineata* in NYC.

### 2.2 Sampling methods

Salamanders were located using extensive visual encounter surveys, where a majority of the accessible cover objects were turned over within the stream reach, as well as cover objects located about 2 m from the water along the stream bank. Individuals were then captured by hand or by dipnet and placed in a gallon‐sized plastic bag. We identified salamanders to species level based on morphology (Gibbs et al., 2007) and recorded SVL (cm; SVL: snout–vent length), weight (g; using a Pesola scale), a GPS coordinate for exact sampling location, and current life stage (larva, juvenile, or adult). We collected DNA from each individual via a small (1–2 cm) tail clip, which was stored in 95% ethanol on site for no more than 12 hr until placed in permanent storage in a −20°C freezer at Fordham University's Biological Field Station (Louis Calder Center in Armonk, NY, USA). All methods of capture and tissue sampling were approved by the Natural Resources Group of the NYC Department of Parks and Recreation (http://www.fws.gov/ventura/species_information/protocolsguidelines/docs/DAFTA), the NY State Department of Environmental Conservation (Permit #1935), and Fordham University's Institutional Animal Care and Use Committee (Protocol No. JMS‐13‐01).

### 2.3 Sampling sites

To examine differences in genetic connectivity across habitats with differing levels of urbanization, we collected tissue samples from *E. bislineata* individuals within streams in an urban, suburban, and rural habitat in the New York City metropolitan area. We classified them as urban, suburban, and rural based on percent impervious surface (respectively, urban = 35.63%, suburban = 2.10%, rural = 0.77%), and human population density (respectively, urban = 198 humans/km^2^, suburban = 38 humans/km^2^, rural = 17 humans/km^2^) within a 12 km^2^ square extent surrounding each habitat. Calculations were performed in ArcGIS 10.3 (ESRI, 2012) using the NLCD Impervious Surface Dataset for 2011 (Xian et al., 2011) with amendments from 2014 (Jin et al., 2013) and the TIGER/Line Shapefile from O'Neil‐Dunne, & Grove (2018).

**FIGURE 1:**
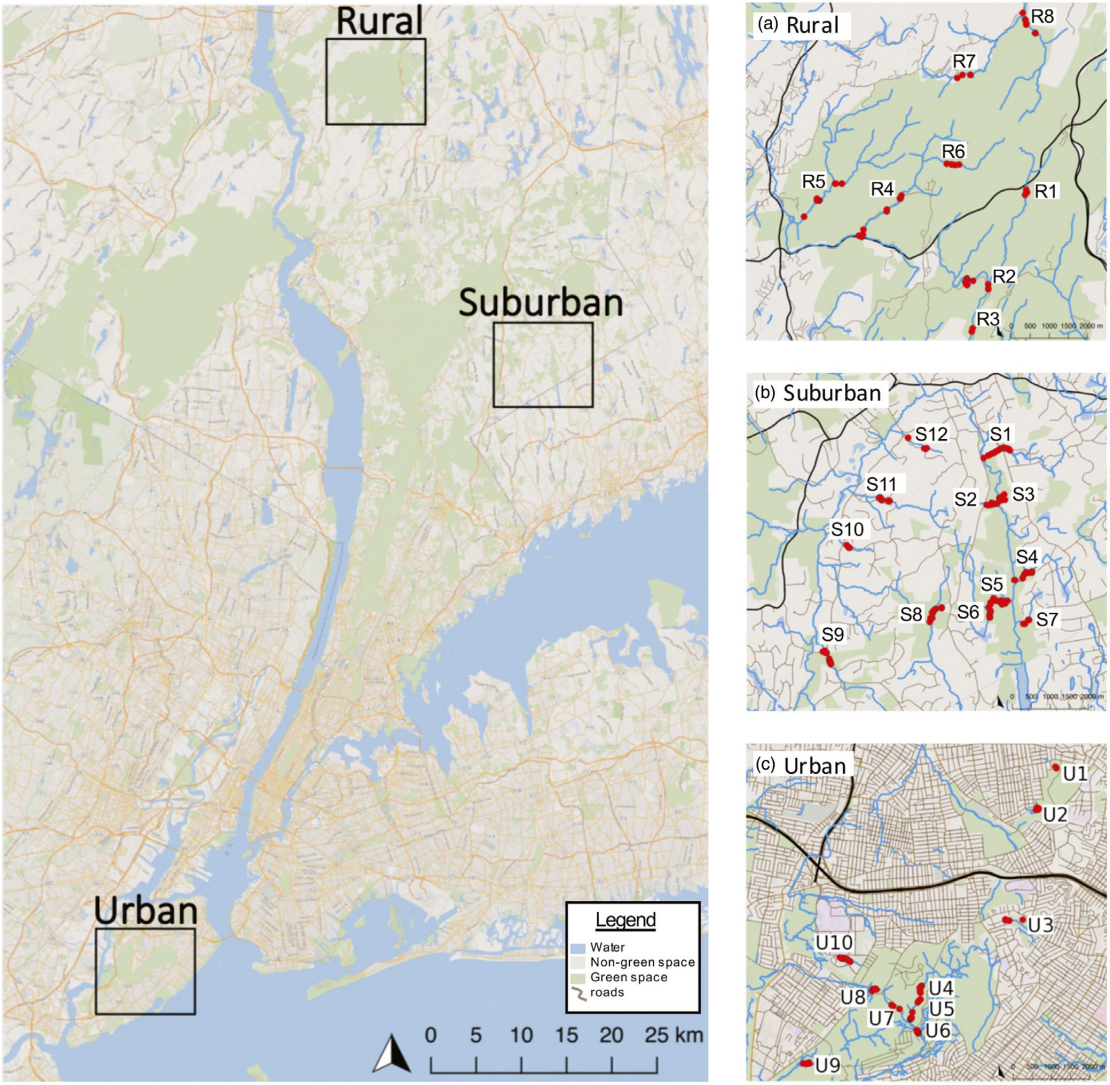
Site map of stream salamander study habitats (*urban*, *suburban*, and *rural*) labeled by location of specific stream sampling site and individual GPS location of each *Eurycea bislineata* sample as red dots across (a) the *rural* habitat in Clarence Fahnestock State Park, Carmel, NY (streams R1–R8), (b) the *suburban* habitat across the Mianus River Watershed, Bedford, NY (streams S1–S12), and (c) the *urban* habitat throughout Staten Island, NY (streams U1–U10). Within the map, black lines indicate primary roadways, thin brown lines indicate secondary roadways, green shades indicate green/open space/parkland, and blue shades indicate water bodies/streams

We collected DNA from 105 *E. bislineata* individuals at ten separate stream reaches within an urban stream network throughout Staten Island, NY, USA. Samples were collected between April 2010 and December 2012 from known locations for this species reported by the NYC Department of Parks and Recreation (*personal communication*). Among these ten streams, half of the study sites were located within forested habitat in the Staten Island Greenbelt State Park, whereas the others were contained within smaller parks or green spaces, and two were located within a golf course (Figure 1c). Many streams were adjacent to or flowing under heavily trafficked roadways.

Between June 2014 and September 2015, we collected DNA samples from 153 *E. bislineata* individuals from 12 stream reaches across a suburban stream network in the Mianus River Gorge Preserve and throughout the surrounding Mianus River watershed in Bedford, NY, USA. Samples were retrieved from accessible streams flowing into the main branch of the Mianus River. Most sites within the Mianus River Gorge Preserve are surrounded by forest and are protected from development. However, some streams on the periphery and those located outside of the preserve are adjacent to large manicured lawns, secondary roadways, and driveways, and one stream flows directly out of a privately owned pond into the Mianus River (Figure 1b).

Lastly, we sampled DNA from 92 *E. bislineata* individuals along eight stream reaches at Clarence Fahnestock State Park in Putnam and Dutchess Counties, NY, USA, from June to September 2017. Samples were retrieved from streams that were accessible within the parklands, where most sites are surrounded by dense forest and undisturbed land (Figure 1c).

### 2.4 SNP genotyping

We isolated and purified *E. bislineata* DNA from approximately 20 mg of tail tissue for each of 351 total samples using Qiagen DNeasy Blood and Tissue Kits (Qiagen, Inc.) with an RNAse treatment and final elution volume of 75 μl. We generated SNP genotypes for all sampled individuals using a double digest restriction site‐associated DNA sequencing (ddRADseq) protocol (adapted from Peterson et al., 2012). We measured the concentration of DNA using either a Qubit 2.0 Fluorometer (Life Technologies) or a Tecan NanoQuant Infinite 200 Pro (Tecan, Inc.) at each step of library preparation. We digested 1,000 ng of genomic DNA for each individual using two restriction endonucleases: SphI‐HF and EcoRI. After digestion, DNA fragments were cleaned with 1.5× Agencourt AMPure XP (Beckman Coulter, Inc., 2013) or Serapure (Faircloth & Glenn, 2011) magnetic beads prepared in the laboratory. Unique barcoded DNA adapters were then ligated to 200–250 ng of digested DNA fragments. Barcoded samples were then pooled into libraries of up to 48 uniquely barcoded samples and purified again with magnetic beads. DNA was then size‐selected for a 376bp–412bp range using a Sage Science Pippin Prep (Sage Science). The size‐selected fragments were PCR‐amplified over 11 PCR cycles with a High‐Fidelity Phusion Polymerase Kit (New England Biolabs) to amplify DNA and to add additional Illumina‐specific index primers to each pool. All PCR products were pooled, cleaned, checked for quality, and quantified with an Agilent Bioanalyzer (Agilent Technologies). The rural and suburban libraries were prepared for separate sequencing lanes and sent out for sequencing using Illumina HiSeq 2000, 2 × 125bp, paired‐end sequencing at the New York University Center for Genomics and Systems Biology. The urban dataset was prepared for a single sequencing lane on an Illumina HiSeq 4000, 2 × 125 bp, paired‐end sequencing at the Translational Genomics Research Institute (TGen).

### 2.5 Bioinformatics

We used STACKS software pipeline version 2.3d (Rochette & Catchen, 2017) for processing the raw sequence reads. *Process_radtags* in the STACKS pipeline was used to sort read pairs by barcode and remove errors from the raw sequencing reads. Reads were then demultiplexed according to their unique barcode adapter and primer index. *Denovo_map.pl* was used to call SNPs and build the RADtag catalog, allowing a minimal number of identical reads in a stack as *m* = *3*, the number of mismatches allowed to merge into one locus as *M* = *3*, and the number of mismatches when building a catalog as *n* = *2*. These parameters were chosen after extensively exploring the parameter space and choosing parameters appropriate for this dataset, based on suggestions from Catchen, Hohenlohe, Bassham, Amores, and Cresko (2013) and Mastretta‐Yanes et al. (2015). We performed this analysis by calling SNPs (running the *Denovo_map.pl* script) on all three datasets compiled together to retain SNPs shared by all three habitats, to ensure our confidence in performing subsequent comparisons between urban, suburban, and rural habitats across the same SNP loci.

Next, we filtered the dataset based on relatedness between individuals as to not bias downstream analyses (Goldberg & Waits, 2010). We used the *‐‐genome* flag in PLINK 1.9 beta (Chang et al., 2015) to filter out individuals with an identity‐by‐descent proportion of greater than 0.5 (full‐sibling or parent–offspring relationship; Anderson & Weir, 2007). By this method, we retained one individual that was part of each closely related pair.

SNPs were further filtered using the STACKS *populations* scripts. We retained only the first SNP per locus (*‐‐write‐single‐snp*) and discarded loci that did not occur in at least 2 out of 3 habitats. We chose to maximize the number of polymorphic loci by retaining only those loci shared by 80% of individuals (*r* = .8; Rochette & Catchen, 2017) and with a minor allele frequency of 5% or greater.

Then, we conducted additional SNP filtering to reduce the number of duplicate loci in the dataset. Plethodontid salamanders have large genomes (~15 to ~47 Gb; *E. bislineata* = ~20.75 GB; Gregory, 2011) composed of many intronic regions with repetitive elements (Rovelli et al., 2018; Sun et al., 2012). Therefore, we followed protocols by Dorant et al. (2020) to identify low‐quality SNPs and removed them from the dataset. These problematic SNPs included duplicate loci (pairs of loci with identical alleles that are most likely a result of paralogous gene duplication), diverged loci (that may be inherited disomically; McKinney, Waples, Seeb, & Seeb, 2017), high coverage loci (that skew the proportion of heterozygotes due to overrepresentation in the dataset), and low confidence loci (that have extreme allele ratios and at least one rare homozygote; Dorant et al., 2020). To remove all of these potentially problematic loci, we started with the filtered VCF file from the STACKS *populations* output and identified low‐quality loci using the *08_extract_snp_duplication_info.py* custom script created by Dorant et al., (2020) available in *stacks_workflow* (https://github.com/enormandeau/stacks_workflow). Then, we used the *10_split_vcf_in_categories.py* script to create a separate VCF file retaining only singleton SNPs to use for subsequent analyses (excluding all other problematic loci mentioned previously). We hereafter refer to this fully filtered SNP dataset as “shared SNP loci,” as these are singleton SNPs shared across habitats and were used for comparative analysis.

Lastly, we reran STACKS *populations* script incorporating the *‐‐whitelist* option (including only singleton SNPs) to recreate fully filtered genepop, structure, and plink files for downstream analyses. We also calculated observed heterozygosity (H_o_), nucleotide diversity (*π*), and pairwise *F*_ST_ (following Weir & Cockerham, 1984) using *populations*. Lastly, we divided individuals into their respective habitat groups (urban, suburban, and rural) to create separate filtered datasets for within‐habitat analyses.

### 2.6 Statistical analyses

To examine population genetic structure, we ran discriminant analysis of principal components (DAPC) using the package *adegenet* in R (Jombart, Devillard, & Balloux, 2010). First, we ran a DAPC using all 351 individuals to examine whether each habitat contained a separate evolutionary cluster of salamanders. Next, we ran separate DAPCs to examine structure within each habitat. In the DAPC program, we used *find.clusters*, which uses Bayesian information criteria (BIC) to reveal the most well‐supported number of clusters present in the dataset. Next, we used *optim.a.score* to understand the ideal number of PCs to retain. DAPC then uses a multivariate approach to partition within‐ and between‐group variation to maximize discrimination between groups. As a complement to these DAPC analyses, we performed principal components analysis (PCA) in the R statistical environment (R Core Team, 2013) using the package *adegenet* (Jombart & Ahmed, 2011) to further explore genetic variation within habitats.

As another way to explore potential clustering, we ran the program ADMIXTURE 1.23 (Alexander, Novembre, & Lange, 2009). This program uses maximum likelihood to estimate individual ancestry proportions and identifies the best‐fitting model based on the number of *K*‐clusters corresponding to the lowest cross‐validation error score (cv‐error). We ran this analysis on all 350 individuals for values of *K* = 3–20 for five iterations at each *K* value. Then, we ran the program separately for each habitat type (urban, suburban, and rural) at *K* = 1–10 for five iterations at each *K* value. Lawson, Van Dorp, & Falush (2018) suggest estimating the “true *K* value” is often complicated by many factors, such as differences in sample size, recent demography, and unsampled ghost populations. Therefore, we explored results across multiple *K*‐values with similar cross‐validation error values.

To test for isolation‐by‐distance (IBD), we performed a standard Mantel test in R using the *ecodist* package (Goslee & Urban, 2007). For this analysis, we calculated a genetic distance matrix based on allelic differences between individuals within each habitat using the *bed2diffs‐v1* program, which is part of the estimated effective migration surface (EEMS) package (Petkova, Novembre, & Stephens, 2015). We looked for patterns of IBD (Sokal, 1979) by comparing the Mantel correlation between the genetic distance matrix and a matrix of Euclidean straight‐line geographic distance between each pair of individuals. We also investigated the distance classes within which the IBD relationship is statistically significant using a Mantel correlogram in *ecodist* (Goslee & Urban, 2007).

Since this species is known to disperse extensively both up‐ and downstream systems (Bruce, 1986; Lowe, McPeek, Likens, & Cosentino, 2008), we also investigated spatial patterns of genetic variation if dispersal is restricted to waterways. We calculated a measure of “isolation‐by‐stream distance” (IBSD; Mullen, Woods, Schwartz, & Sepulveda, 2010) to investigate whether there was a correlation between genetic distance (allelic differences between individuals) and geographic distance through the freshwater stream network. To explore these patterns, we calculated a straight‐line distance through linear waterways using the R package *riverdist* (Brauer, Unmack, Smith, Bernatchez, & Beheregaray, 2018). Since the waterways within the urban habitat are not all connected, the samples from stream sites in the far north (U1 and U2), and site U10 were excluded from this analysis. To calculate this distance, we used the National Hydrography Dataset's (U.S. Geological Survey, 2011) linear hydrology shapefile (for the suburban and rural habitats), and the NYC Parks Stream Hydrography Mapping stream layer (for the urban habitat; NYC Parks & Recreation, 2019) to create connected waterway networks for each habitat. After projecting the GPS locations for each sampled individual onto these networks, we calculated the Euclidean geographic distance between each individual along the connected waterway. Lastly, we calculated the correlation between pairwise genetic distance and pairwise geographic stream distance using a standard Mantel test in the package *ecodist* (Goslee & Urban, 2007). Afterward, we compared the Mantel *R* values from the stream distance analysis (IBSD) versus overland Euclidean distance analysis (IBD) to assess the relative importance of in‐stream versus overland gene flow.

To explore spatial patterns of the genetic data, and to visualize a representation of population structure within each habitat, we created estimated effective migration surface (*EEMS*; Petkova et al., 2015). First, we calculated genetic differentiation between individuals for each habitat using the *bed2diffsv‐1* function and used this genetic distance matrix and spatial coordinates for each individual as input to run the *EEMS* analysis. We used *EEMS* to estimate whether there is more or less migration between neighboring demes (discrete populations) than predicted by an isolation‐by‐distance model. The total area over which we performed the analysis was a 12 km^2^ extent surrounding each habitat type. We ran *EEMS* multiple times for each habitat, starting with the default hyperparameters, then fine‐tuned the proposal variances until the proposals were accepted ~20%–30% of the time (as suggested in Petkova et al., 2015). We also started with the default number of MCMC (Markov chain Monte Carlo) iterations and increased this number, the burn‐in, and the number of thinning iterations until the model converged. We ran the models at several deme values (a grid between which we can calculate genetic similarity over an area) and presented the results at 800 demes. The EEMS output visualizes geographic regions where genetic similarity is higher or lower than expected under an isolation‐by‐distance null model. Lastly, we created Moran's eigenvector maps (MEMgene), in the *memgene* package in R (Galpern, Peres‐Neto, Polfus, & Manseau, 2014) to account for the influence of spatial effects on genetic structure (Manel, Poncet, Legendre, Gugerli, & Holderegger, 2010) to detect fine‐scale spatial patterns of genetic differences between individuals within the urban, suburban, and rural habitats.

## 3 RESULTS

### 3.1 Across all individuals

**FIGURE 2:**
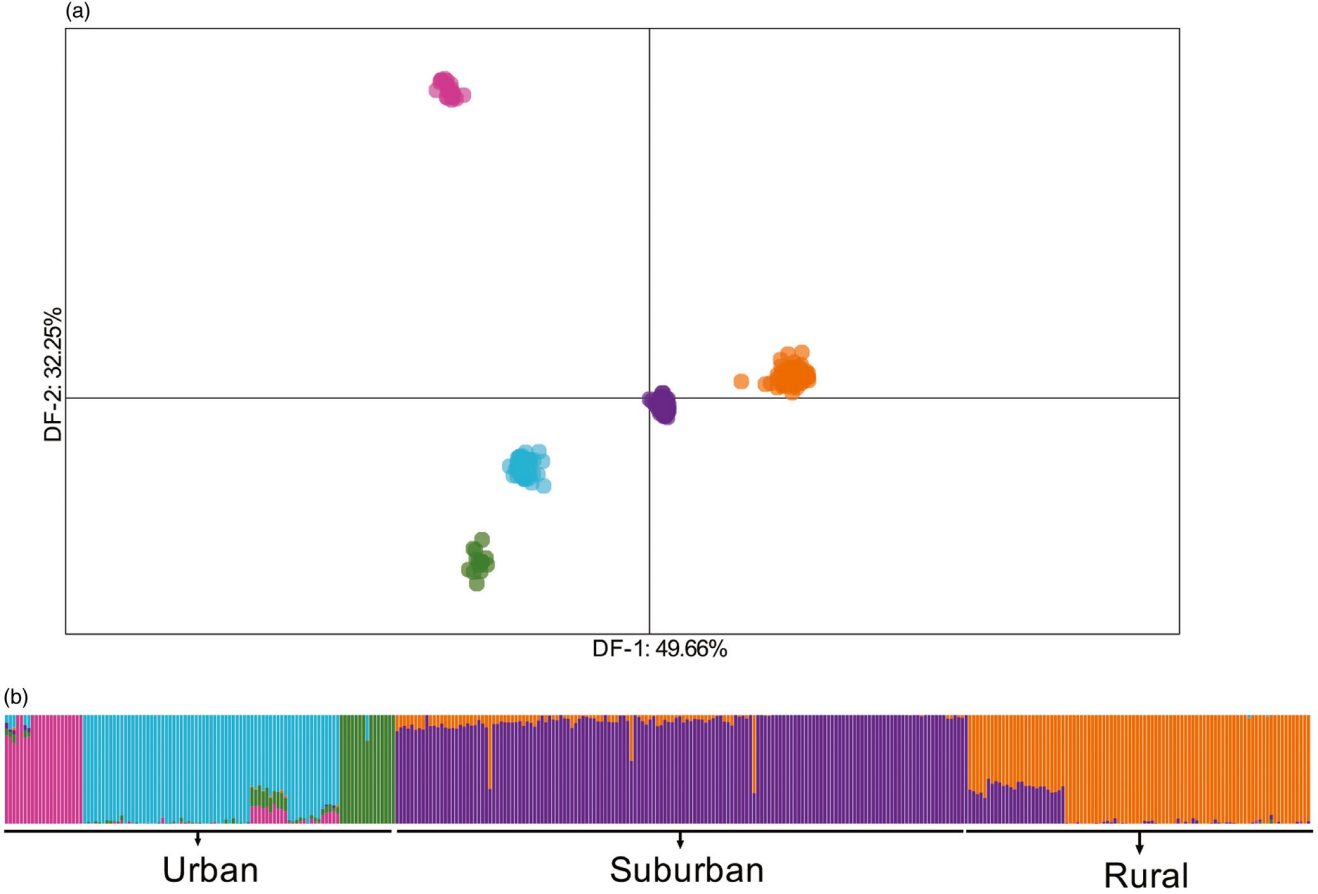
(a) Discriminant analysis of principal components shown for discriminant function DF‐1 (capturing 49.66% of variation in the dataset) and DF‐2 (capturing 32.25% of the variation). (b) ADMIXTURE bar plot comparing all individuals. Each vertical bar on the *x*‐axis represents an individual which are grouped by *urban*, *suburban*, and *rural* habitats. The *y*‐axis represents the proportion of ancestry represented as different colors. The bar plot is represented at *K* = 5, which had the lowest cross‐validation error in the analysis

After sorting and quality filtering using STACKS and subsequent filtering for duplicate loci, the final dataset contained 15,314 SNP loci. Results from STACKs showed the greatest genetic differences were observed between urban versus suburban habitats (*F*_ST_ = 0.110), followed closely by differentiation between the urban versus rural habitats (*F*_ST_ = 0.095). The lowest genetic differentiation was between the suburban and rural habitats (*F*_ST_ = 0.038). The population summary statistics revealed similar levels of genetic diversity (*H_O_* and *π*) across the three habitats (*H*_O_ = 0.265–0.278 and *π* = 0.272–0.304). The urban habitat contained the greatest number of private alleles, suggesting greater population structure within this habitat (Table 1). The DAPC analysis among all 351 individuals revealed distinct evolutionary clusters for the urban, suburban, and rural habitats. The first discriminant function (DF) of the DAPC distinctly separated the urban from the suburban/rural individuals. The second DF showed separation between the suburban and rural clusters (Figure 2a) and additional structuring among urban stream sites. The ADMIXTURE analysis across all individuals supported the DAPC results, with *K* = 5 as the most well‐supported model (lowest cv‐error value = 0.496; Figure S2A), with each habitat as a distinct evolutionary cluster and additional structure within the urban habitat (Figure 2b).

**TABLE 1:** (A) Population genetic statistics for *Eurycea bislineata* across the *urban*, *suburban*, and *rural* study habitats calculated with 15,314 shared SNP loci. The data below include the following: *N*, the sample size; *N_e_*, the mean effective population size calculated over 5 random sets of 5,000 SNPs; *Private alleles*, the number of private alleles in that dataset; *H_O_*, observed heterozygosity; *π*; nucleotide diversity; and *F*_IS_, the inbreeding coefficient. (B) Weir–Cockerham *F*_ST_ values between the *urban*, *suburban*, and *rural* habitats

(A)						
Habitat	*N*	*N*_e_	Private alleles	*H*_o_	*π*	*F*_IS_
Urban	105	54.240	1,791	0.265	0.304	0.117
Suburban	154	1,025.700	315	0.278	0.272	−0.014
Rural	92	1,020.100	385	0.275	0.290	0.041

(B)			
	Urban	Suburban	Rural
Urban	—	0.110	0.095
Suburban	—	—	0.038
Rural	—	—	—

### 3.2 Within the urban habitat

**FIGURE 3:**
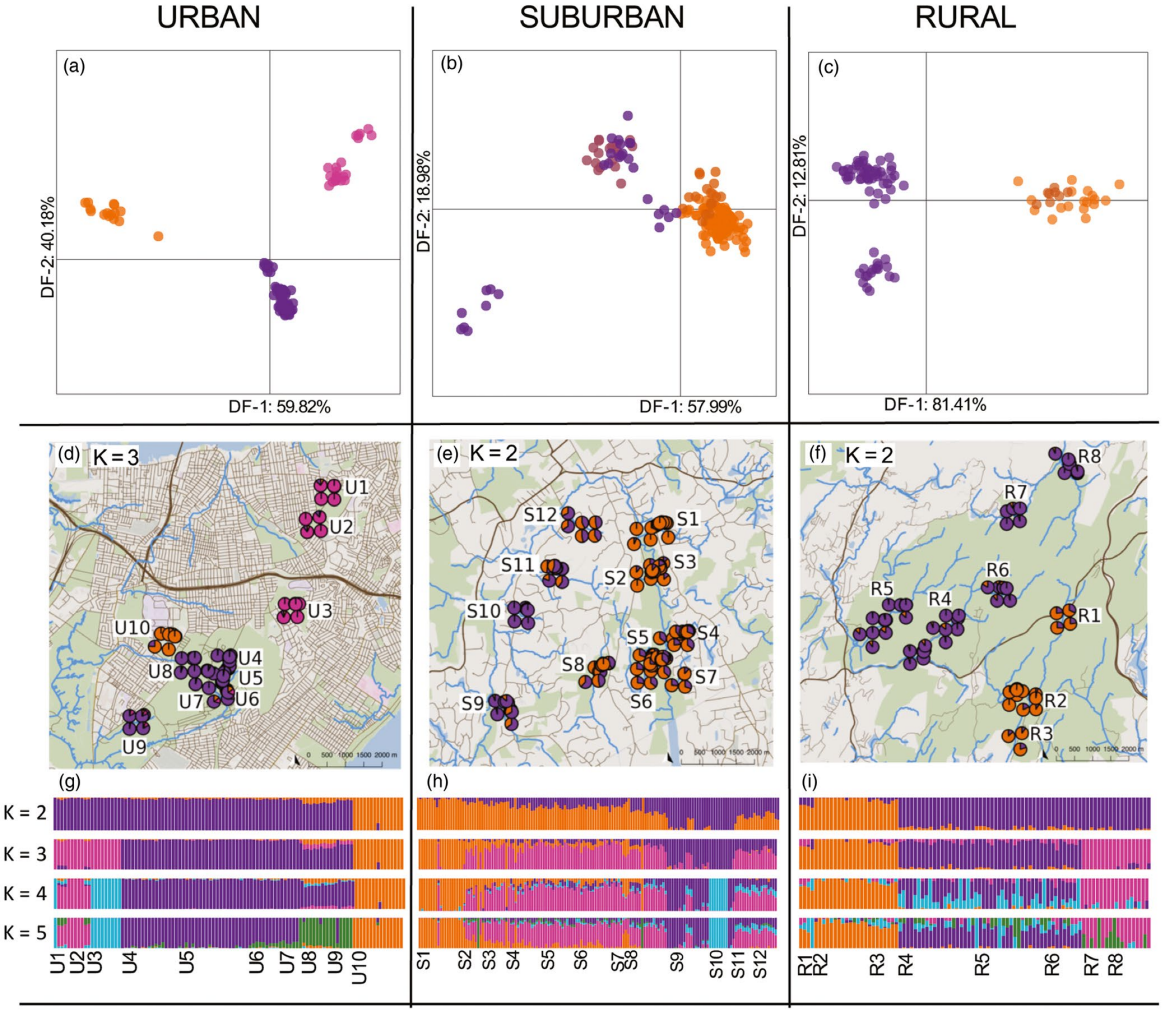
Comparison of the *urban* (first column), *suburban* (second column), and *rural* (third columns) datasets across three different visualizations of population admixture and structure; discriminant analysis of principal components (top row), mapped ADMIXTURE ancestry proportions (middle row), and ADMIXTURE bar charts (bottom row). (a–c) Each DAPC is shown for discriminant function DF‐1 (horizontal) and DF‐2 (vertical) axes. (d–f) ADMIXTURE ancestry proportions are shown as pie charts and are displayed graphically (green on the map indicates open/greenspace, blue lines indicate stream/river systems, bold brown lines indicate primary roadways, and thin brown lines indicate secondary roadways). The urban dataset is shown on the map at *K* = 3 (the most well supported), whereas the suburban and rural datasets are shown graphically at *K* = 2 (although their most well supported was *K* = 1). (g–i) Below each map, ADMIXTURE bar plots are displayed at *K*‐values 2 through 5, where each vertical bar on the *x*‐axis represents individuals labeled by stream site below, and the *y*‐axis represents the proportion of ancestry represented as different colors

The urban dataset contained 105 individuals with an average of 18.3× coverage across SNP loci. The DAPC analysis identified *K* = 3 (with the lowest *BIC* value) as the most well‐supported number of evolutionary clusters in the dataset. The DAPC showed three distinct genetic clusters for *E. bislineata* in streams within the urban habitat. The first DF (60% of overall variation) emphasized genetic differences between stream site U10 (orange) and stream sites in the north (U1‐U3; pink; Figure 3a). The second DF (40% of overall variation) identified an additional cluster including stream sites located within the Staten Island Greenbelt protected area (U4‐U9; yellow) compared to other streams outside that region. The PCA supported these results with distinct clustering between these same stream sites (Figure S1A). The cross‐validation likelihood scores in ADMIXTURE analysis also indicated *K* = 3 (Figure S2B) as the highest likelihood for number of evolutionary clusters among urban streams (shown in a graphical pie charts on a map at *K* = 3; Figure 3d). This analysis was congruent with the DAPC (and PCA) in how it grouped the respective streams into separate clusters. At *K* = 3, there was a slight signature of admixture for site U8 within the Staten Island Greenbelt Park and the most southern site U9 with mixed ancestry from both northern (pink) and western (orange) genetic signatures. Additional k‐means clustering options were explored for this analysis (*K* = 2–5), since their cross‐validation scores did not vary greatly from the most well‐supported *K* = 3 cv‐value (Figure S2B). The *K* = 4 model distinguished site U3 (blue) from streams U1 and U2 (pink), and the *K* = 5 model separated stream sites U8 and U9 (green; Figure 3g).

**FIGURE 4:**
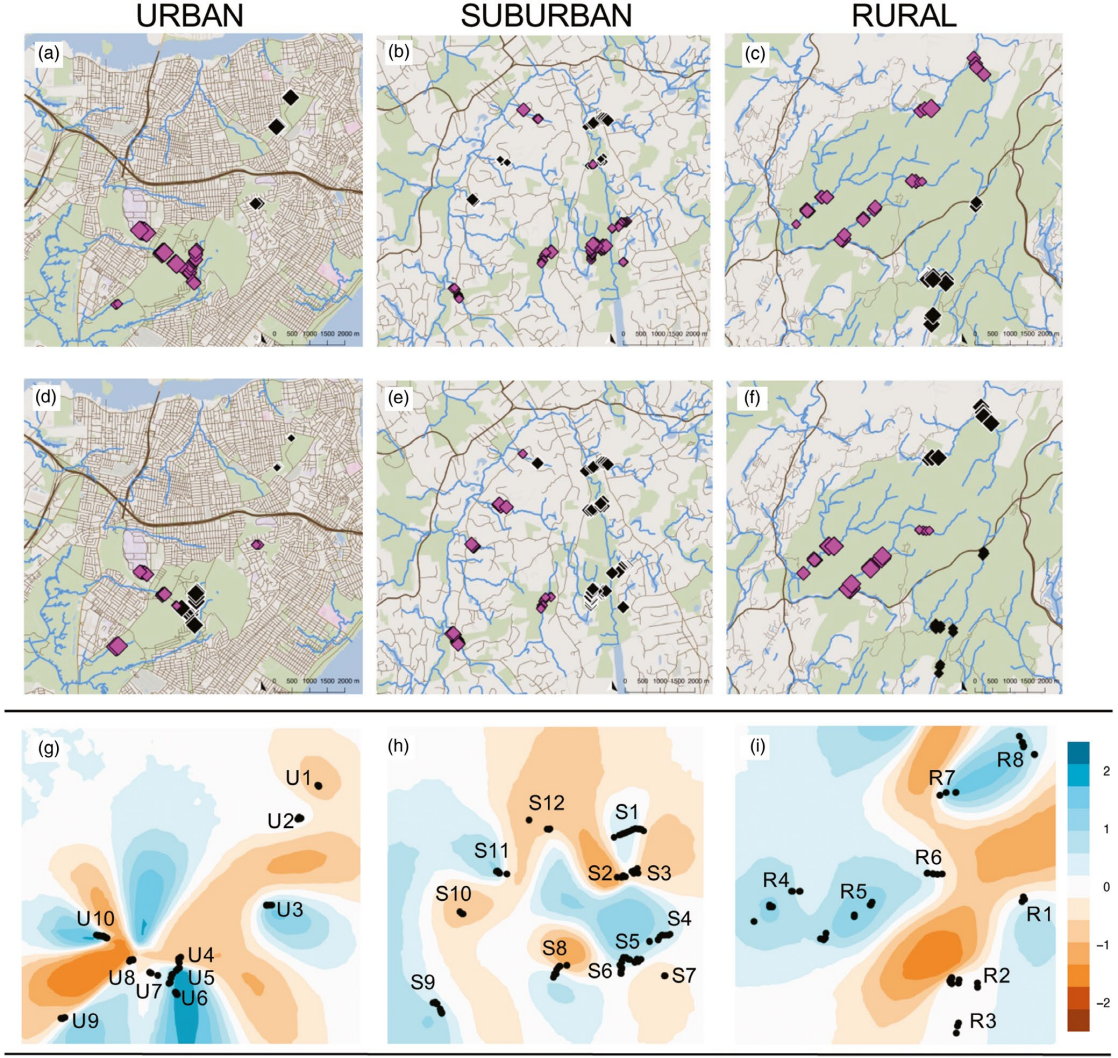
MEMgene analyses overlaid on a map for the *urban* (first column; a & d), *suburban* (second column; b & e), and *rural* (third column; c & f) habitats presented at two different axes; MEMaxis‐1 (top row; a–c) and MEMaxis‐2 (bottom row; d–f). Locations of individuals are represented by pink diamonds (negative values) and black diamonds (positive values) at varying sizes indicating the magnitude of genetic distance (based on *bed2diffs* genetic distance measure). Estimated effective migration surfaces for (g) the *urban*, (h) *suburban*, and (i) *rural* habitats where darker shades of blue indicate greater than expected migration (0–2), darker shades of orange indicate less than expected migration (−2 to 0), and white indicates the null hypothesis of isolation‐by‐distance (0). The solid black dots indicate locations of sampled individuals labeled by stream sampling location

The MEMgene analysis, accounting for spatial autocorrelation and the geographic location of samples, showed results congruent with the clustering analyses, where the first MEMaxis separated the more northern urban sites (U1‐U3) from the remaining sites (U4‐U10; Figure 4a). The second MEMaxis revealed an east/west divide between stream sites U1‐U5 and U8, and stream sites U7, U9, and U10 (Figure 4d). Estimated effective migration surface (EEMS) analysis revealed low migration between multiple stream sites located throughout the urban habitat. It revealed a dark band of orange (area of reduced migration) surrounding and differentiating site U10 from the rest of the urban stream sites (U1–U9; Figure 4g). Another narrow band of dark orange (representing lower than expected migration) was apparent in the middle of the streams located within the Staten Island Greenbelt Park property, with reduced migration between sites U6 and U7. Both areas showed greatly reduced migration despite their proximity to other neighboring stream locations. This map also revealed areas of reduced migration (orange color) between southwestern stream sites (U4–U10) and those in the north (U1–U3), complementing results from the clustering analyses. Lastly, this analysis also showed limited migration between northern sites (U1–U3) that may be due to IBD (appearing in white) rather than IBB (appearing in orange). The only sampled area with greater than expected migration (a band of darker blue) was within the same stream reach (U4/U5; Figure 4g) located centrally in the urban habitat.

**FIGURE 5:**
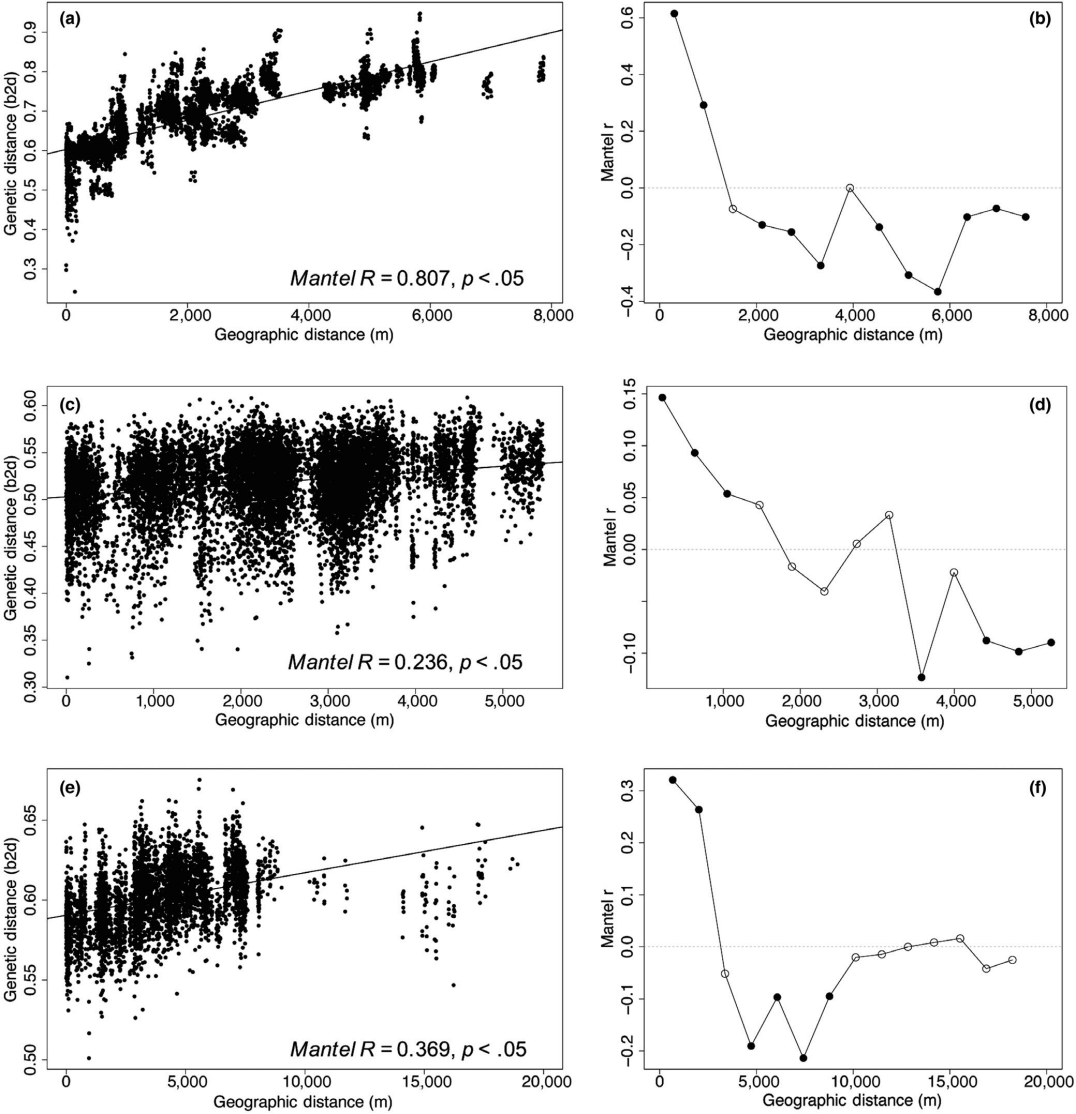
The standard Mantel test (first column a, c, e) with Euclidean geographic distance (m) on the *x*‐axis and genetic distance (*bed2diffs* genetic distance measure) on the *y*‐axis. The correlation between geographic and genetic distance is statistically significant for the (a) *urban,* (c) *suburban,* and (e) *rural* datasets. The second column includes Mantel correlograms with Euclidean geographic distance (m) on the *x*‐axis and Mantel *R* on the *y*‐axis for (b) the *urban*, (d) *suburban*, and (f) *rural* dataset, where black circles indicate significant spatial autocorrelation, and white circles indicate nonsignificant spatial autocorrelation over different (Euclidean) geographic distance classes (m)

The correlation between genetic and geographic distance was significant (Mante*l R* = .807, *p* < .05), showing IBD across the urban habitat (Figure 5a). The mantel correlogram indicated that this correlation is no longer significant at a distance beyond approximately 900m (Figure 5b). Isolation‐by‐stream distance (IBSD) also resulted in a significant effect within the urban habitat (Mante*l R* = .820, *p* < .05). In this urban habitat, both distance over land and distance along the path of a waterway are correlated with genetic differences between individual stream salamanders (Figure S3A).

### 3.3 Within the suburban habitat

The suburban dataset included 154 salamanders with an average of 12.1× coverage across shared SNP loci. The DAPC for the suburban habitat (Figure 3b) revealed less distinct clustering than was observed for the urban habitat, with the lowest *BIC* value indicating *K* = 1 clusters in the suburban habitat. The first DF (58% of overall variation) separated clusters located on the eastern half (S1–S8; orange) and western half (S9–S12; purple) of the suburban sampling area. The second DF also highlighted genetic differences (with less support; 19% of total variation) across western stream sites, showing genetic differences between individuals sampled within a single stream site, S10, from the rest of the stream sites in the west (purple). The ADMIXTURE cv‐likelihood scores showed the best support for a *K* = 1 model (Figure S2C). We showed ancestry proportions in pie charts within a map at *K* = 2, which reflected the slight east/west divide we saw from the DAPC results (Figure 3e). We explored models with values of *K* between 2 and 5, which showed overall greater admixture, or mixed ancestry, across these *K*‐values in the suburban habitat (Figure 3h) as compared to the distinct clustering assignment seen over all *K*‐values in the urban habitat (Figure 3g). *K* = 3 revealed differentiation of site S1 (orange) from those streams to the south (pink), and *K* = 4 displayed further substructure between the sites within the west where S10 (blue) separated as its own distinct cluster. As we increased up to *K* = 5, additional admixture was added to the ancestry of individuals within the eastern streams (S2–S7; Figure 3h).

MEMgene analyses mirrored the clustering results, where the second MEMaxis revealed differentiation on an east/west axis (Figure 4e). EEMS analysis also supported substructuring results from the ADMIXTURE analysis, that is, a large area of orange (less than expected migration) between multiple streams in the western portion of the suburban habitat (Figure 4h). We also saw stream S1 surrounded by a band of orange (region of reduced migration), reflecting the results from ADMIXTURE at *K* = 3 (Figure 3h). Lastly, there was greater than expected migration (blue) occurring between streams S4–S6 within the least disturbed area of the preserve property (Figure 4h).

There was a weak, yet statistically significant IBD relationship within the suburban habitat (Mantel *R* = .233*, p* < .05; Figure 5c). The Mantel correlogram showed this correlation was only significant within the first distance class, up to 975 m, but not beyond (Figure 5d). Isolation‐by‐stream distance (IBSD) was not significant within the suburban habitat (Figure S3B).

### 3.4 Within the rural habitat

The rural habitat included 92 individual salamanders with an average of 9.2× coverage across the shared SNP dataset. The DAPC analysis indicated the lowest *BIC* value was *K* = 1 cluster for the rural habitat. The first DF (81% of overall variation) from DAPC showed genetic clustering between streams in the southeastern portion of the sampling area (R1‐R3; orange) separated from those located more northwest (R4–R8; purple; Figure 3c). The second DF (with weak support; 12% of the total variation) exhibited further differentiation of the northernmost streams R7 and R8 from the more centrally located streams R4‐R6 (Figure 3c).

Despite the distinct clustering we saw in the ADMIXTURE bar plots (Figure 3i) for rural streams, the most well‐supported model for the rural habitat was *K* = 1 evolutionary cluster (lowest cv‐value; Figure S2D). We again explored higher *K*‐values, where *K* = 2 (depicted graphically on the map; Figure 3f) reflected the major trend seen in the DAPC, distinct clustering between northwestern streams (R4–R8; purple) and southeastern streams (R1–R3; orange). *K* = 3 showed the most northern stream sites R7 and R8 separated as their own cluster (pink). At higher values of *K* = 4 and *K* = 5, there was no more additional structure, only the addition of admixture to the more centrally located stream sites R4–R6 (Figure 3i).

MEMgene results for the rural habitat supported the clustering analyses, where the first MEMaxis showed genetic differentiation in northern and southern stream sites (Figure 4c). For the second MEMaxis, there were genetic differences detected along an east/west divide (Figure 4f). EEMS analysis showed greater than expected migration (blue) occurring across the centrally located rural streams R4‐R6 (Figure 4i). This was the largest geographic area of greater than expected migration across all three habitats. Yet despite this habitat being the most rural, a large band of orange (representing less than expected migration) was found between the most northern sites (R7 and R8) and the stream sites directly south (R1–R3) with another band of dark orange, representing reduced migration between the southeastern sites (R1–R3) and those located centrally (R4–R6). These regions of reduced migration supported the results from the first MEMaxis (Figure 4c), and clustering at *K* = 2 (Figure 3f).

Isolation‐by‐distance was determined to significantly affect spatial genetic structure of *E. bislineata* individuals within the rural habitat (Mante*l R* = .369, *p* < .05; Figure 5e). The IBD correlation was significant over a greater distance, up to 2 km in the rural habitat (Figure 5f), as compared to only about 1km in the suburban and urban habitats. IBSD was also significant for the rural habitat (Mante*l R* = .419, *p* < .05, Figure S3C).

## 4 DISCUSSION

The results from this study revealed a difference in genetic structure for *E. bislineata* salamanders occupying urbanized habitats compared to salamanders in suburban and rural habitats in the NYC metropolitan area. Multiple analyses indicated that urban salamander populations had lower genetic connectivity between streams compared to nonurban populations. Contrary to our prediction, urbanization and loss of connectivity did not affect overall levels of genetic diversity across habitats. This finding conflicts with other studies that show reductions in gene flow decrease genetic diversity due to fewer immigrants bringing new alleles into the population (Delaney et al., 2010; Vandergast, Bohonak, Weissman, & Fisher, 2006). Yet our findings did reveal a smaller effective population size in the urban compared to the suburban and rural habitats signifying a future potential threat to maintenance of genetic diversity. Spatial patterns of genetic differentiation were significantly affected by IBD in all habitats, although a weaker pattern in the suburban and rural habitats suggests limited dispersal across the urbanized habitat than elsewhere. IBSD analysis revealed that in‐stream dispersal also influenced gene flow in the urban and rural habitats, yet gene flow through streams was not significantly affected by stream distance in the suburban habitat. Additionally, regions with low connectivity correlating with potential barriers (IBB) were also detected in all habitats, specifically in areas containing some type of urban development or disturbance. Previous studies on stream salamanders revealed that urbanization reduces salamander abundance and occupancy in urban areas (Barrett & Price, 2014; Hamer & McDonnell, 2008; Price, Browne, & Dorcas, 2012; Price, Dorcas, Gallant, Klaver, & Willson, 2006). The present study demonstrates that urbanization also influences genetic connectivity for *E. bislineata*. Our findings agree with others reporting reduced genetic connectivity due to urbanization among a variety of vertebrate taxa with varying life histories and dispersal strategies (deer; Fraser, Ironside, Wayne, & Boydston, 2019; puma; Trumbo et al., 2019; fish; Blanton, Cashner, Thomas, Brandt, & Floyd, 2019; salamanders and frogs; Homola, Loftin, & Kinnison, 2019).

### 4.1 Urbanization reduces genetic connectivity between streams for *E. bislineata*

Dispersal is the primary mechanism driving gene flow, where reproductive mode and physiological requirements are directly related to dispersal (Lourenço, Antunes, Wang, & Velo‐Antón, 2018). For an amphibian, the ability to disperse through the landscape and successfully reproduce is often directly linked to the location and availability of water sources (Semlitsch, 2008). *E. bislineata* rely on headwater streams and moist forest habitat, and thus are predisposed to exist in patchy mosaics where conditions between stream branches strongly influence population dynamics (Nelson‐Tunley, Morgan‐Richards, & Trewick, 2016). Overall, the complexity and multidimensionality of stream salamander ecology and their reliance on highly specific local conditions will determine their presence and success in urban areas. Results from the present study show that *E. bislineata* populations can persist within an urban matrix, but dispersers that connect subpopulations are affected by urbanization.

Collectively, our analyses demonstrate that both geographic distance and urban disturbance affect gene flow. Evolutionary clustering analyses revealed low connectivity across areas with potential barriers between urban stream populations including roadways, commercial organizations, and residential housing. Roadways also influence genetic structure in brown frogs (*Rana japonica*) in Japan (Kobayashi et al., 2018), and housing, industry, and roadways act as barriers to gene flow in the endangered growling grass frog (*Litoria raniformis*) in Australia (Hale et al., 2013). Richardson (2012) and McCartney‐Melstad, Vu, and Shaffer (2018) found both distance and roads shape the genetic structure of wood frogs (*Lithobates sylvatius*) and Eastern tiger salamanders (*Ambystoma tigrinum*), which are often sympatric with *E. bislineata* in northeastern North America. Generally, our results are congruent with other studies that show urbanization affects spatial and population dynamics, as well as genetic structure for a broad range of amphibians (Jean‐Marc et al., 2018; Emel, Olson, Knowles, & Storfer, 2019; Marsh et al., 2008; Munshi‐South et al., 2013; Scheffers & Paszkowski, 2011; Vanek, King, & Glowacki, 2019; Villasenor, Driscoll, Gibbons, Calhoun, & Lindenmayer, 2017) and other animal taxa (DeCandia et al., 2019; Jaffé et al., 2019). Mechanisms underlying the effects of urbanization on amphibian gene flow include physical barriers to dispersal, lack of free‐standing water or moist microhabitats, and the presence of extensive light and noise pollution (Eigenbrod, Hecnar, & Fahrig, 2009; Hale et al., 2013). The cumulative effects of continued urbanization can potentially alter how species interact with the landscape thus affecting functional connectivity between populations.

Urbanization does not have to be extreme to cause fragmentation of natural habitat or confer negative effects on populations of native species. Moderate suburban development decreases canopy cover and increases water temperatures in streams (Holgerson, Lambert, Freidenburg, & Skelly, 2018), and leaves patches of unsuitable terrestrial habitat interspersed among undeveloped, seminatural areas (Hitchings & Beebee, 1997). These variables may have a direct impact on stream salamander populations which persist within suburban waterways and disperse overland. Other studies report reduced occupancy and abundance with increased housing density for *E. bislineata* in exurban areas (periphery of suburbs leading to more rural habitat) in Connecticut (Macklem, Helton, Tingley, Dickson, & Rittenhouse, 2019). Our study showed rather high levels of genetic connectivity between suburban streams, suggesting remnant seminatural landscape features may support connectivity across this suburban habitat. Remaining natural areas in an urban or suburban matrix can, at times, provide corridors for gene flow (Aleixo‐Pais et al., 2018; Furman et al., 2016; Llorens, Ayre, & Whelan, 2018). For example, remnant vegetation in fragmented peri‐urban areas aids connectivity between populations of Australian quenda (*Isoodon obesulus*; Ottewell et al., 2019). Even the most unlikely sources of habitat, such as golf courses, can maintain enough suitable waterbodies and green corridors to maintain connectivity between populations of amphibians (Saarikivi, Knopp, Granroth, & Merilä, 2013) and reptiles (Winchell & Gibbs, 2016). In our study, residential lawns and patches of forest may be able to provide ample habitat to maintain genetic connectivity across a suburban area with moderate levels of human disturbance.

Dozens of studies have now shown that urban development increases genetic differentiation between populations for many noncommensal species (Johnson & Munshi‐South, 2017). The results of this study on *E. bislineata* in NYC support the “urban fragmentation” prediction made by Miles et al. (2019), where urbanization impedes gene flow leading to greater genetic differentiation between populations. On the contrary, the results presented here do not support the accompanying prediction for genetic diversity, where urban fragmentation does not contribute to the loss of genetic variation at presumptively neutral loci. What we find here for *E. bislineata* bolsters their conclusion that variation in life history traits and heterogeneity in the landscape/city complicates whether, and to what degree, urbanization affects neutral genetic variation across taxa. Theoretically, one immigrant per generation (an average of one immigrant every 10 generations) can maintain genetic diversity in the receiving population (Lowe, Kovach, & Allendorf, 2017), thus blurring the degree to which urban barriers can affect gene flow. Our use of genomic methods for an animal with such a large genome (Rovelli et al., 2018) will help to make informed decisions about the evolutionary potential for population persistence of a common salamander species and be able to extrapolate the risks posed to more rare, related species. McCartney‐Melstad et al. (2018) recently uncovered previously undetectable genetic structure in Eastern tiger salamander populations on a small scale in Long Island using a genomic dataset compared to a previous study using microsatellite markers. Our study adds to the growing literature using genomic data to study urban evolution and will further understanding of how neutral evolutionary processes such as genetic drift and landscape ecology can affect population dynamics of native species (Munshi‐South et al., 2016).

### 4.2 *E. bislineata* populations maintain genetic diversity despite urbanization

A reduction in genetic diversity may hinder evolutionary potential in changing environments (Hand, Lowe, Kovach, Muhlfeld, & Luikart, 2015). Many studies have found that urban populations are less genetically diverse than rural populations for a variety of amphibian species (Hitchings & Beebee, 1997; Noël & Lapointe, 2010; Noël, Ouellet, Galois, & Lapointe, 2007). Our results do not fit this pattern, as we found nearly equivalent levels of genetic diversity (*H_O_* and *π*) among the urban, suburban, and rural habitats. Similar to our findings, a study on fire salamanders (*Salamandra salamandra*) in Oviedo, Spain, showed genetic differentiation between urban populations was not associated with substantial losses in genetic diversity (Lourenço, Álvarez, Wang, & Velo‐Antón, 2017). Despite human disturbance, amphibian populations are already at risk of having lower genetic diversity due to their life history strategies, patchy distribution, and risk of population size fluctuation with rainfall fluctuation (Allentoft & O'Brien, 2010). Pan et al. (2019) hypothesized that the maintenance of high levels of genetic diversity across populations of Chinese torrent frog (*Odorrana tormota*) may be due to larger population sizes and a lack of significant bottlenecks. In this study area in NYC, immigration from neighboring populations may supplement local population size and genetic variation, making these populations less prone to extinction. Yet our results showed greatly reduced effective population size in the urban compared to the suburban and rural habitats, signifying that these urban stream salamander populations may experience the effects of genetic drift and inbreeding depression in future generations.

In this study, there were logistical limitations of the time available for sample collection. Samples were collected over multiple years across the different habitats, which may have a confounding effect on how allele frequencies change over time. This time constraint was due to the feasibility of collection of a small, cryptic, and difficult to catch salamander species, and limited human power in collecting 351 tissue samples across a large geographic range. This was especially true across the urban habitat where access to sites with lower stream salamander density resulted in a longer period of sample collection (2 years) to obtain enough DNA samples for robust analysis. Some studies state that genetic diversity is in a transient state, but after a peak the rate of decay is a slow process (Alcala, Streit, Goudet, & Vuilleumier, 2013). However, by using reduced representation genome sequencing, the estimates of genetic structure and genetic diversity measures incorporating allelic differences are robust, because measures were calculated across thousands of loci across the genome. Any allelic changes incurred over a few generations are not expected to greatly change the results unless there has been a major, rapid population expansion or contraction. Since urbanization has been established across this study area (NYC) for decades, it is reasonable to assume that the current rate of habitat alteration was not any greater than historic disturbance regimes, and thus likely had a consistent effect across sample collection dates in the results reported here.

### 4.3 Exploratory analyses lead the way to future landscape genetic studies

Inferences from population genetic studies indicate diverse responses for amphibians living in urban landscapes (Scheffers & Paszkowski, 2011), but these analyses lack the ability to specify particular environmental and anthropogenic barriers or variables that cause resistance to gene flow. The type of analyses used in this study only shows how genetic breaks correlate with landscape variables that may be influencing gene flow between populations in these habitats. For example, genetic differentiation we detected between stream sites located above and below a major roadway in the rural habitat could be a result of roadway itself but also could be due to these waterways existing in separate watersheds. Future landscape genetic analyses could identify the specific variables, either natural and/or anthropogenic, affecting gene flow for stream salamanders in both terrestrial and aquatic landscapes. We know that natural barriers to gene flow, such as slope (Lowe, Likens, McPeek, & Buso, 2006), and anthropogenic barriers such as reduction of forest cover (Cecala et al., 2014; Emel et al., 2019) and stream impoundments (Kirchberg, Cecala, Price, White, & Haskell, 2016) can affect in‐stream dispersal and abundance of salamanders. Therefore, future studies should incorporate landscape genetic modeling approaches to assess which natural or anthropogenic variables more strongly influence gene flow for *E. bislineata* in these urban, suburban, and rural habitats in the NYC metropolitan area.

## 5 CONCLUSION

Populations of amphibians are declining worldwide, including even previously common species (Bank et al., 2006), especially in areas affected by urbanization (Cecala et al., 2018; Price et al., 2012; Price, Muncy, Bonner, Drayer, & Barton, 2016). We presented evidence that urbanization can prevent stream populations from exchanging sufficient individuals to avoid genetic drift even in a species that is relatively common and highly abundant, such as *E. bislineata*. In NYC, despite the close proximity of some green spaces, overland habitats may be too heavily urbanized for dispersal and gene flow to regularly occur. These fragmented areas can promote inbreeding, which can be exacerbated by population declines due to reductions in habitat quality. Overall, this study reaffirms the need to maintain connected greenspace (Jackson & Fahrig, 2016) between neighboring streams as well as connectivity within streams to sustain salamander populations, even for common species. Continued urbanization that reduces gene flow between populations of native species living in cities and moderately developed suburban areas will alter their evolutionary trajectory and the viability of future populations.

## CONFLICT OF INTEREST

None declared.

## Supporting information

Fig S1‐S3

## Data Availability

Data for this study are available at the National Center for Biotechnology Information (NCBI) Sequence Read Archive (SRA), accession number PRJNA633375.
