# Supplementary material for: Urbanization reduces gene flow but not genetic diversity of stream salamander populations in the New York City metropolitan area
Source: Evol Appl. 2020 Jun 12;14(1):99–116. doi: 10.1111/eva.13025 (PMC7819553; doi:10.1111/eva.13025)
Supplement: Supplementary file 1 — Fig S1‐S3 [file EVA-14-99-s001.docx]

**Appendix S1.**

**Figure S1**. Principal Components Analysis results for the **(A.)** *urban,* **(B.)** *suburban,* **(C.)** and *rural* datasets.

**Figure S2**. ADMIXTURE proportion of cross-validation error (cv) on the y-axis, shown across different values of *K* on the x-axis for **(A.)** *all individuals,* **(B.)** the *urban*, **(C.)**, *suburban*, **(D.)** and *rural* habitats.

**Figure S3**. Connected waterway maps created for isolation-by-stream-distance analysis (IBSD) with sampling localities (blue squares) mapped along the waterway (connected multi-colored stream sections) for the **(A.)** *urban*, **(B.)** *suburban*, **(C.)** and *rural* habitats. Significant Mantel test figures for the results of IBSD are shown for the **(D.)** *urban* and **(E.)** *rural* habitats (suburban habitat not shown because IBSD was not statistically significant). Standard Mantel figures include geographic distance along the waterway on the x-axis (m) and genetic distance (*bed2diffs* genetic distance) on the y-axis.

C.

B.

A.

A.

D.

C.

B.

URBAN

SUBURBAN

ALL INDIVIDUALS

RURAL

D.

A.

*Mantel R* = 0.820, *p* < 0.05

B.

E.

C.

*Mantel R* = 0.419, *p* < 0.05
